# Haematological Malignancies in Systemic Sclerosis Patients: Case Reports and Review of the World Literature

**DOI:** 10.1155/2017/6230138

**Published:** 2017-05-04

**Authors:** M. Colaci, D. Giuggioli, C. Vacchi, C. Ferri

**Affiliations:** Rheumatology Unit, Azienda Ospedaliero-Universitaria Policlinico di Modena, University of Modena and Reggio Emilia, Modena, Italy

## Abstract

*Background*. The association of systemic sclerosis (SSc) and haematological cancers was reported in a large number of case reports and cohort studies, describing SSc patients with highly heterogeneous clinical pictures.* Objective*. We reviewed the literature to better describe SSc patients with haematological malignancies.* Methods*. SSc cases complicated by haematological malignancies described in the world literature were collected; other 2 cases referred to our centre were reported.* Results*. One hundred-thirty SSc subjects were collected from 1954 up to date. The mean age of patients at cancer diagnosis was 56.1 ± 16.7 years; 72% of patients were females. In 60% of cases, the diagnosis of haematological malignancy was described within 5 years of SSc diagnosis. In 7.8% of cases, coexistence of Sjögren's syndrome or other autoimmune disorders was cited. Sixty-six cases with lymphoma (in the majority of cases B-cell neoplasms), 28 with leukaemia (chronic lymphocytic form in 9), 14 with multiple myeloma plus one solitary IgM plasmocytoma, and 16 with myeloproliferative disorders were found. No specific SSc subsets seem to be related to haematological malignancies.* Conclusions*. We remarked the importance of clinical work-up in SSc, in order to early diagnose and treat eventual occult haematological malignancies, especially during the first years of the disease.

## 1. Introduction

The association between malignancies and connective tissue diseases was widely reported in literature [1]; namely, systemic sclerosis (SSc) has showed relatively high incidence of lung, breast (contrasting data), and haematological cancers, as demonstrated by meta-analysis on population-based cohort studies [2, 3]. However, these studies usually reported the frequencies of specific malignancies in the course of SSc, without further characterization of the patients. In this respect, the subset of “haematological tumours” included different types of malignancies that generally were not described in detail. On the other hand, a large number of case reports may be found in literature [4–54], describing SSc patients with highly heterogeneous clinical pictures complicated by the onset of cancer. These reports could potentially contain detailed data that are not included in registry or large cohort studies [55–67].

The haematological neoplasms originate by the myeloid or the lymphoid cell lines, historically named leukaemias or lymphomas, based on the prevalent location in the blood or the lymph nodes, respectively. Afterwards, more than 70 nosological entities were identified, classified according to the 2008 World Health Organization (WHO) classification of neoplasms of the hematopoietic and lymphoid tissues, on the basis of the recognition of distinctive features in terms of morphology, clinical picture, immunophenotype, and genetic and molecular characteristics [68]. Indeed, the systematic categorization of the haematological malignancies evolved during the decades from the Rappaport classification of 1966 to the WHO classification in 2001 [69], lastly updated in 2016 [70], transposing the new knowledge achieved in the field of histology and cytogenetic/immunohistochemical profiling of malignancies.

In these lights, the known association between SSc and haematological malignancies should be better characterized, especially as regards eventual associations between SSc features and specific type of blood neoplasms. Moreover, specific SSc subsets might be associated with peculiar haematological cancers, besides other comorbid predisposing conditions. Therefore, we aimed to collect all SSc cases complicated by haematological malignancies described in the world literature, searching eventual specific clinical patterns.

## 2. Patients and Methods

We analysed the whole SSc patients' cohort, recruited in our Rheumatology Centre, including 454 cases referred to our university-based hospital from 1 January 2003 to 31 December 2016, in order to find the patients who presented haematological neoplasms in their clinical history, after SSc diagnosis. For all patients, detailed clinical records were available, which eventually comprehended the documentation regarding the haematological diseases.

Secondly, the electronic databases, including PubMed, Embase, Scopus, Web of Science, SciELO, J-Stage, and Google Scholar, were searched for studies that described the cases with the association between haematological cancers and SSc, including all available previous articles and non-English reports. Search terms were “systemic sclerosis” or “scleroderma” and “h(a)ematological cancer” or “lymphoma” or “leuk(a)emia”. Myeloproliferative disorders (search terms: “myelofibrosis”, “chronic myelogenous leukaemia”, “polycythemia vera”, “essential thrombocytemia”) were also considered, as well as “MGUS”. All available data included in the published studies were analysed, evaluating all the information useful for patients' clinical profiling.

We considered all SSc patients who developed blood cancers; otherwise, the cases describing SSc onset* after* the diagnosis of haematological cancer were excluded. Likewise, the studies that did not exactly indicate the timing of SSc and cancer diagnoses were excluded. On the contrary, the patients with contemporary or very close onset of the two pathologic conditions (blood cancer diagnosis within 1 year from SSc diagnosis) were registered as patients with “probable paraneoplastic” syndrome. Finally, we did not consider studies regarding morphea or localized scleroderma nor sclerodermiform syndromes following antineoplastic treatments.

## 3. Case Reports

### 3.1. Case 1

**Figure 1:**
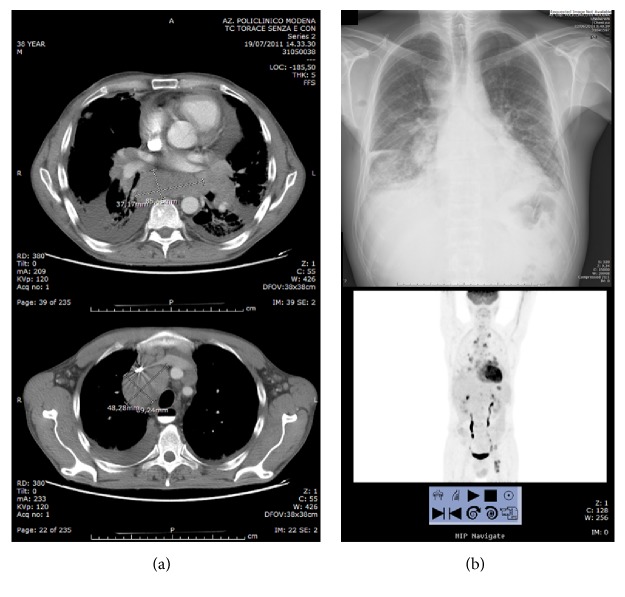
Radiological chest studies of our patient number 1. (a) Two scans of high-resolution CT, showing the mediastinal adenopathic masses. (b) Standard X-ray and total body PET scan, showing diffuse high-metabolism adenopathies.

A nonsmoker, 35-year-old male patient referred to our centre in 2008 and received a new diagnosis of SSc. The onset of the disease was few months before, featured by skin thickening of face, hands, forearms, and chest; skin ulcers or calcinosis was absent. He complained of Raynaud's phenomenon, fatigue, and mild sicca syndrome. No anti-topoisomerase I or anti-centromere autoantibodies were found (ANA 1 : 640 speckled), while anti-Ro/SSA and anti-SSB were present (secondary Sjögren's syndrome). He presented typical oesophageal dyskinesia and interstitial lung disease with reduction of the forced vital capacity to 69% and of the lung diffusion for the carbon monoxide to 33%. Heart function was normal, and no signs of pulmonary hypertension were found, while a slight increase of creatine-kinase was reported. At chest high-resolution CT (HRCT) performed within the first year of follow-up, an enlargement of the thymic shape emerged; the histology confirmed the presence of thymic hyperplasia; thus, the gland was removed, also hoping to counteract SSc progression [71]. After 2 years, the patient presented a deterioration of dyspnea with dry cough, extension of skin thickness to the whole body, increased fatigue, mild fever, and body weight loss. A further chest HRCT revealed a new mediastinal enlargement due to adenopathy (Figure 1); primary mediastinal B cell lymphoma (a type of diffuse large B cell non-Hodgkin lymphoma, NHL) was diagnosed by means of video-assisted thoracoscopic surgery (VATS) lymph node biopsy. The standard CHOP-R (cyclophosphamide, hydroxydaunorubicin, vincristine, prednisone, and rituximab) regimen was chosen; however, after few months, before any clinical improvement, the patient died from sepsis.

### 3.2. Case 2

A 72-year-old woman referred to our centre in 2012 from another hospital, where she was followed for SSc. The disease's onset dated back to 28 years before. Among SSc features, we emphasize the presence of pulmonary arterial hypertension, treated with sildenafil and, successively, ambrisentan. Calcinosis, telangiectasias, sclerodactyly, and anti-centromere antibodies, but not dysphagia nor interstitial lung disease, were found. As comorbidity, the patient presented severe lower limb arteriopathy obliterans, responsible for digital gangrenous lesions. Moreover, patient's history was marked by the diagnosis of low-grade tubular breast carcinoma in 2006; then, she underwent right quadrantectomy. Six years later, X-ray scan revealed a 2 cm pulmonary opacity in the right lower lobe. After chest CT confirmation, lobectomy was performed; the histological analysis diagnosed an extra-nodal marginal mature B cell lymphoma (BALToma). Given the absence of metastasis, no radio-/chemotherapy was considered necessary after the lung resection. To date, the patient is doing well, without presenting recidivism.

## 4. Review of the Literature

Table 1 summarized all cases of SSc patients complicated by haematological malignancies found in literature [4–67]; the studies that do not give any information about SSc features and/or haematological cancer types were excluded. Both case reports and cohort studies were included, even though, usually, only the first ones reported complete description of the clinical histories. To the best of our knowledge, 130 (including our 2 cases) subjects affected by SSc and haematological cancer were collected, from the first case described in 1954 up to date. Majority of patients were from Europe and USA and were Caucasian, while 18% of persons were of Asian ethnicity, coming from the Far Eastern Countries. The mean age of patients was 56.1 ± 16.7 years, without gender difference as regards age, with the higher prevalence of cases in the sixth decade. 72% of the cases were women. The diagnosis of haematological malignancies was frequently close to SSc diagnosis: indeed, in about 30% of cases, scleroderma could be considered as “probable paraneoplastic,” while for other 30% of patients the cancer was diagnosed within 5 years of SSc diagnosis. Sporadic observations of blood cancer were also reported during the further years, even after several decades.

Among SSc patients with haematological malignancies, the diffuse skin subset was reported in 28% of cases. As regards serology, anticentromere and anti-Scl70 autoantibodies were equally found; of note, a relevant percentage (29%) of specific anti-nuclear autoantibodies (ANA) was observed. Organ SSc involvement was not frequently described; anyway, no peculiar associations may be found, because the malignancies could be observed both in SSc patients with diffuse skin involvement and interstitial lung disease and in the “CREST” patients' subset. In a few cases, overlapping Sjögren's syndrome (a disease with well-known increase of haematological cancer risk) was suspected or clearly reported; moreover, rheumatoid arthritis was recognized in 2 patients, porphyria cutanea tarda in other 2, and pemphigus vulgaris in 1. Finally, only in few patients a detailed clinical history was available, giving the possibility of identifying other eventual cancer risk factors (i.e., smoking).

As regards haematological malignancies, we collected 66 cases with lymphoma (including our 2 cases), 28 with leukaemia, 14 with multiple myeloma (plus one solitary IgM plasmocytoma), and 16 with myeloproliferative disorders; in 3 the exact diagnosis was not expressed. Many types of lymphoma were reported, often not better specified than the mere definition of “non-Hodgkin lymphoma”; in the other cases, the diffuse large B cells lymphoma was the most frequent (8 cases plus 1, our case). With the exception of few patients, all lymphomas described are classifiable as mature B cells neoplasms; in fact, we registered only 3 lymphomas of T cells and 2 from histiocytic cells, while 6 cases of Hodgkin's lymphoma were found.

Considering the 28 cases developing leukaemia, 9 patients showed the chronic lymphocytic form. T cells leukaemia was present in only 2 persons; 8 cases were not better specified. Finally, among myeloproliferative disorders, 4 cases of myelofibrosis and 11 of chronic myelogenous leukaemia were found; just one patient presented polycythemia vera, while no cases of essential thrombocythemia were found.

The clinical courses of the treated patients may be divided into 2 prognostic patterns, substantially equivalent in percentage: (1) rapid improvement up to remission, often associated with SSc features amelioration; (2) rapid deterioration until death from infectious causes. The latter pattern was invariably observed in patients over 50 of age; no other clinical features useful for prognostic purposes were found.

## 5. Discussion

**Figure 2:**
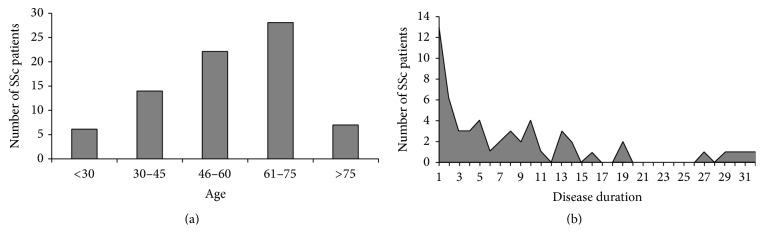
Distribution of SSc patients with haematological malignancies on the basis of age at tumour diagnosis (a) and SSc disease duration (b).

It is known from the literature that the incidence of haematological malignancies is significantly increased in SSc [1–3]; in this review, we tried to better define this statistical association gathering together all SSc cases complicated by blood cancers previously described. We found that the majority of cases presented the B cells non-Hodgkin lymphoma (especially the diffuse large B cells lymphoma, as well as our case number 1), the multiple myeloma, and the chronic lymphocytic leukaemia; furthermore, also myeloproliferative disorders were frequently described in the course of SSc. Besides the heterogeneity of the cases reported in literature, we found that the diagnosis of haematological neoplasms was a precocious event in SSc patients' clinical histories, particularly within 5 years of SSc diagnosis in the majority of cases (Figure 2). Therefore, given the possibility of successful treatments for a potentially aggressive disease, the clinical-serological surveillance for haematological malignancies in SSc patients should be addressed.

Regarding the demographic characteristics of the SSc patients with blood cancers, we found a higher frequency of males (28%) in comparison to the female/male ratio previously described in large SSc case series [72], probably because of the higher NHL incidence among male subjects [73].

Even though anecdotal in several patients, we underline the coexistence of other autoimmune disorders or pathologic conditions known for their increased risk of cancer. Indeed, the omission of relevant anamnestic information in the descriptions of patients reported in literature is presumable, especially for the registry/large cohort-based studies designed for the statistical analysis of cancers epidemiology. As regards serology, the presence of a specific ANA in SSc patients with haematological cancers was found in 29% of cases, more than generally reported in previous cohort studies [72]. In this respect, Altintas et al. [74] detected ANA in more than 20% of 179 patients affected by lymphomas, even though the majority of them did not show autoimmune diseases. Furthermore, in an elegant study by Guyomard et al. [75], 347 NHL patients and 213 controls were investigated by means of indirect immunofluorescence technique on Hep2 cells. ANA were significantly more frequent in the first group (19% versus 5.6%), before any treatment, particularly in presence of follicular or mantle B cell lymphomas. The latter are characterized by a high rate of cells proliferation and a large number of apoptotic cells, leading to the exposition of large amount of nuclear antigens, eventually targeted by patients' immune system.

The association between autoimmune diseases and haematological neoplasms is an intriguing question; indeed, more than 10% of lymphoid malignancies occur in the setting of an autoimmune disorder [76]. Accumulated evidences indicate that autoreactive B cells are more prone to undergo malignant transformation. In parallel, the chronic activation of the inflammatory response due to autoantigen-driven immune stimulation in specific organ tissues (i.e., the parotid gland in Sjögren's syndrome) is associated with an increased risk of lymphomas [76, 77]. Furthermore, the epidemiological data from large population studies on other autoimmune diseases, such as rheumatoid arthritis or systemic lupus erythematosus, showed a high risk of B cells haematological neoplasms, particularly the diffuse large B cell lymphoma [78, 79]. Consistently, in SSc, we found a clear-cut prevalence of B cells versus the T cells cancers, suggesting that the systemic autoimmune activation plays a pivotal role in the carcinogenetic evolution.

Besides the evidences of pathogenetic and statistical links between SSc and haematological malignancies [1–3], the exact mechanism responsible for cancers is not understood. In other autoimmune disorders specific etiologic factors (i.e., HCV for mixed cryoglobulinemia [80]) lead to persistent stimulation of the immune system and, eventually, to lymphomagenesis. Differently, SSc etiology remains obscure, despite the probable role of a few infectious triggers able to chronically infect immune cells [81]. Overall, a deeper knowledge of the SSc etiopathogenetic processes, probably different for several disease's subsets, could help to better quantify the risk for different types of cancers, including haematological neoplasms.

Since the majority of patients described in the present study developed the tumours in the first years of the disease (Figure 2), including our first case, the possible iatrogenic effect of immunosuppressors may be easily excluded. On the contrary, it was hypothesized that the immune alterations in the early phase of SSc present a different pattern, which tends to change during the disease's follow-up [82].

We previously demonstrated a high prevalence of thymic hyperplasia in SSc patients, particularly during the first years of the disease [71]. Given the fundamental role of the thymus in the maturation of T lymphocytes, it might be assumed that a pathological alteration of the thymic microenvironment could lead to a deficient or incomplete T cell maturation, which might have a role in the immunological alterations of SSc etiopathogenesis. Nonetheless, the autoreactivity of T cells strictly involves also B cells that produce a number of different autoantibodies, which in turn stimulate fibroblasts' toll-like receptors-4 [83] and induce endothelial dysfunction [84]. B cell infiltrates may be detected in SSc patients' skin or in affected areas of the lungs, so giving the rationale for the therapeutic use of rituximab [85]. Therefore, even though T cells are considered the driving force of the autoimmune pathogenesis in SSc, it is not surprisingly the observation of an increased risk for B cells-derived malignancies.

Upon the onset of autoimmune responses, lymphoid tissues undergo histological changes due to the remodelling of the tissue architecture in parallel with the phenotypic transformations of immune cell populations. Recently, Sangaletti et al. [76] hypothesized that an erroneous remodelling of the stromal microenvironment in secondary lymphoid organs could facilitate malignant transformation of lymphocytes, in presence of persistent immune stimulation. Thus, lymphomagenesis would be a result of disrupted myeloid and lymphoid function in lymphoid tissues that harbour autoreactive proliferating T and B cells. In this respect, our paradigmatic case developed a lymphoma that probably rose in the thymus, which was histologically disrupted by the preexistent thymic hyperplasia.

In SSc patients, several studies demonstrated the activation status of the peripheral B lymphocytes with an impaired percentage of apoptotic cells compared to healthy controls [86]. Moreover, Wang et al. [87] found that the levels of histone acetylation and methylation (responsible for increased gene transcription) in B cells from SSc patients correlate with disease activity. Furthermore, serum concentrations of BAFF and APRIL, cytokines regulating B cell activity, survival, and proliferation, are found elevated in SSc in comparison with healthy controls, particularly in patients with active or severe disease [86]. In this light, we might assume that the sclerodermic patients with poorly controlled disease (an occurrence more probable in the early phase of SSc, like in our first case) are more prone to develop haematological malignancies in their clinical histories.

In SSc, the increase of B cells survival and activation sounds apparently in contrast with the finding of augmented Fas (CD95) expression on the surface of memory B cell that facilitate Fas-mediated apoptosis. However, the incessant loss of these lymphocytes is coupled to the increased production of naive B cells and plasma cells in order to maintain B cell homeostasis [88]. Therefore, the amplification of the percentage of less mature B lymphocytes understandably leads to a major risk for lymphoid carcinogenesis.

Finally, as opposite scenario, we briefly mention the possibility that cancer mutations might trigger SSc itself, at least in patients with anti-RNA polymerase III autoantibodies [89]. Neoplasms could harbour missense mutations in the gene coding for the polymerase III polypeptide A, leading to the production of an altered protein. The latter could stimulate an immune response and, possibly, a cross-reaction against the normal protein; this immune response could be relevant in the pathogenesis of a subset of SSc [90].

The present study shows a few limitations. Firstly, even though this review included the higher possible number of studies, several cases of SSc complicated by blood cancers described in literature were lost [91, 92], because of the unavailability of the necessary information for the purposes of our study. This limitation may be exceeded only with further studies designed ad hoc, including large case series.

Secondly, our review included a number of case reports, in which contemporaneous SSc and haematological malignancy are more likely to be reported than cases where the diagnoses are far apart. In particular, SSc patients who suffered from haematological malignancies longer after SSc onset were more unlikely to be published because it was difficult to emphasize the relationship of ‘SSc and haematological malignancies.' However, also SSc cohort studies seem to confirm the higher incidence of blood cancers in the first years of SSc. Anyway, the eventual exposure to immunosuppressive therapy in SSc patients with longer disease duration could be considered a further risk factor for cancer development.

In conclusion, SSc may be complicated by several types of cancers, including haematological malignancies. More frequently, B cells-derived lymphomas and leukaemias may be diagnosed in the first years of the disease and represent a significant warning for patients' prognosis. To date, no specific SSc features could predict which subjects present major risk for blood cancer; thus, a careful surveillance of SSc patients should be addressed.

## Figures and Tables

**Table 1:** Haematological diseases in course of systemic sclerosis.

First author/year	Number cases	Study type (country)	Age/sex	Dis. duration	Skin subset	Serology	Visceral inv.	Ass. Sjogren	History notes	Clinical picture	Hematological malignancy	Outcomes
Agard/2000	1	CR (France)	62 F	14	L	ACA	None	No	MGUS	Spleno/lymphoadenop., ascites	Small B cell NHL	Improved with CHOP

Airo'/2011	1	CS (360 Italian pts)	nd	nd	nd	ACA	nd	nd	nd	nd	NHL	nd

Alacacioglu/2005	1	CR (Turkey)	57 M	3	nd	nd	nd	nd	nd	Bilateral upper/lower eyelid hernias	Orbital marginal zone NHL	Improved with chemo/radiotherapy

Angeli/1991	1	CR (France)	42 F	4	L	ACA	nd	No	nd	Splenomegaly	CLL	nd

Arai/2009	1	CR (Japan)	31 F	1	nd	nd	nd	nd	nd	None	Thymic large B-NHL	Remission with CHOP

Arnaud/2006	1	CR (France)	76 F	11	L	nd	E	nd	*H. pylori* +	nd	Gastric MALT lymphoma	nd

Bachleitner-Hofmann/2002	1	CR (Austria)	73 F	14	L	ACA	L, E	nd	MGUS	nd	MM	Marked and sustained improvement with therapy for MM and SSc

Baldini/1994	1	CR (Italy)	59 F	1	nd	ANA	nd	nd	nd	nd	Lymphocytic Ly of intermediate diff.	Improved

Bellis/2014	1	CR (France)	37 M	1	L	ANA	nd	nd	nd	Right axillary lymphoadenopathy	CD30+ anaplastic Ly	Lymphoma and SSc remission with BMT

Ben Ghorbel/2005	1	CR (Tunisy)	70 F	6	L	Scl70	L	No	nd	Generalized lymphoadenopaties	Follicular B NHL	Improved with CHOP

Bielefeld/1996	5	CS (21 French pts)	39 F, 56 F, 69 F, 12 M, 71 M	0, 6, 6, 9, 2	nd	nd	nd	nd	nd	nd	CML, AML, immunocytoma, Burkitt's Ly, Waldenstrom d.	nd

Bistue/1990	1	CR (Argentina)	36 F	nd	D	nd	L	No	nd	Dyspnea, splenomegaly, and fever	Myelofibrosis	nd

Cavallero/1994	1	CR (Italy)	79 M	nd	D	ANA	nd	nd	Carpenter	Purpura of legs	Hairy cell leukemia	Died for pneumonia after 3 months

Charlanne/2004	1	CR (France)	72 F	<1	L	ACA	No	Yes	Overlap RA-SS	Neutropenia and lymphocytosis	Large granular lymphocyte leukemia	Sustained (>1 year) improvement with MTX 7.5/week for leukemia and autoimmunity

Chatterjee/2005	5	RS (538 US pts)	2 NHL are F	nd	2 NHL : 1 L, 1 D	nd	nd	nd	nd	nd	NHL (2); MM (2); leukemia (1)	nd

Čolović/2011	1	CR (Serbia)	55 F	20	L	nd	nd	nd	nd	Intense facial pruritus, paraproteinemia	MM	Remission for SSc and MM

Comer/1992	1	CR (UK)	31 F	1	L	ANA	E, L, H	No	nd	Neck/mediastinum lymphadenopathy	IIb-staged HL	HL remission (MOPP), SSc evolution by 1 year

Constans/1993	1	CR (France)	65 F	0	L	ACA	CREST	No	nd		Hairy cell leukemia	nd

Derk/2003	1	CR (USA)	66 M	2	D	Scl70	E	No	nd	Expanding mass at the tongue base	Large B-NHL	Remission with CHOP

Doyle/1985	5	CS (USA)	10; 22; 31 54; 70 F	4; 9; 9; 40; 57	L	nd	CREST	nd	nd	nd	HL; MM (2); “malignant Ly”; CLL	Variable outcomes

Duggal/2002	1	CR (India)	42 M	nd	nd	nd	nd	nd	nd	nd	HL	nd

Duncan/1979	7	CS (2,141 USA pts)	50–79 F	2, 0, 1, 3, 0, 61, 1	nd	nd	nd	nd	nd	nd	CLL (3), MM, lymphosarcoma (2), CMML	Died by 1 year (2), alive > 5 years (4)

Dupond/1989	1	CR (France)	73 F	nd	L	ACA	CREST, L	Yes	nd	Splenomegaly	CMML	nd
Ferroir/1991	1	CR (France)	42 M	2	nd	ANA	nd	No	nd	nd	Mixed follicular Ly	Diagnosis at autopsy

Frigui	1	CR (France)	56 F	10	L	Scl70	L, K	No	nd	Skin lesion	Cutaneous B-cell Ly (supraorbital)	Regression after radiotherapy but relapse

Gisser/1979	1	CR (USA)	29 F	4	nd	nd	L, H	nd	Previous chlorambucil treat.	Anemia	CML	Died for bronchopneumonia

Hall/1978	1	CR (USA)	22 F	14	nd	nd	E calcinosis	No	Generalized lipodystrophy	Diffuse lymphoadenopathies	Nodular sclerosing HL	nd

Hasegawa/1999	1	CR (Japan)	43 M	<1	D	ANoA	nd	No	nd	Neck/armpits lymphoadenopathies	Diffuse large T cell NHL	Lymphoma and SSc remission (CHOP 4 cycles)

Haviv/1997	1	CR (Israel)	72 F	1, 5	L	ANA	L, K	No	nd	Fever, wasting, and arthralgias	Diffuse small cell NHL	Death for sepsis

Hill/2003	2	RS (441 Australian pts)	F	nd	nd	nd	nd	nd	nd	nd	Not better specified	nd

Hoshida/2004	7	CS (Japan)	57 (56–65), 2/5 M/F	2.2 (0–12)	nd	nd	nd	2/7	nd	nd	HL (2); diffuse large B cell Ly (5)	All died by 1 year

Kaşifoğlu/2006	1	CR (Turkey)	50 F	7	L	Scl70	L	SSA+	nd	Weakness, weight loss	CML	Improved with HU

Kaşifoğlu/2016	3	CS (340 Turkish pts)	nd	nd	nd	nd	nd	nd	nd	nd	MM, CML, follicular NHL	nd

Katz/1979	1	CR (USA)	57	11	nd	ANA	nd	nd	Pemphigus v.	nd	Diffuse histiocytic Ly	nd

Kyndt/1997	1	CS (123 French pts)	76 F	8	nd	Scl70	L	Yes	nd	nd	CMML	nd

Kojima/2006	2	CS (Japan)	nd	nd	nd	nd	nd	nd	nd	nd	B cell follicular Ly	nd

Kuo/2012	6	RS (2,053 Taiwanese pts)	1 M, 5 F	nd	nd	nd	nd	nd	nd	nd	Ly (3), myeloprolif. dis. (2), CML (2)	nd

Lee/2001	1	CR (Korea)	56 F	15	L	ACA	CREST	No	Porphyria c.t.	Splenomegaly	Myelofibrosis	nd

Marto/2014	1	CR (Portugal)	76 F	0	L	ACA	L	No	Multiple polyps of the colon	Multiple adenop., diarrhea, and rectorrhagia	IIIb-staged mantle cell NHL of the colon	Ly remission with R-CHOP

Miyamoto/2000	1	CR (Japan)	55 F	17	nd	nd	nd	No	nd	Fever, fatigue, pancytopenia, and splenomegaly	Myelofibrosis	Treated with pulse steroids and transfusions

Olesen/2010	18	RS (2,040 Danish pts)	M/F 9/9	2/18 : <1	nd	nd	nd	nd	nd	nd	NHL (10); leukemia (7)	nd

Owlia/2014	1	CR (Iran)	58 M	15	L	nd	E	No	smoker (30 p-y)	Lumbar pain (extensive bony infiltration)	MM	Death 2 years after VAD/bortezomib

Ozturk/2006	1	CR (Turkey)	54 F	5	L	nd	CREST	No	nd	Sweet syndrome	Myelofibrosis	Improved with steroids and hydroxyurea

Parma/1996	1	CR (Italy)	68	3	nd	nd	nd	nd	nd	Primitive muscle and bone involv.	Large multilobated B-cell NHL	Improved

Prochorec-Sobieszek/2004	1	CR (Poland)	22 F	<1	L	PmScl	nd	No	nd	Parotid swelling	Parotid MALToma	nd

Rodrigues/1989	1	CR (Brazil)	nd	nd	nd	nd	nd	nd	Concom. thyroid adenoca.	nd	Ileal B-cell Ly	Rapid deterioration until death

Rosenthal/1993	3	RS (233 Swedish pts)	nd	<1 (1)	nd	nd	nd	nd	nd	nd	NHL (2), not better specified hematological cancer (1)	nd

Rothfield/1992	1	CS (148 USA pts)	nd	nd	nd	Scl70	nd	nd	nd	nd	Lymphocytic Ly	nd

Roumm/1985	3	CS (262 USA pts)	33 F, 50 F, 71 F	3.5, 6.5, 5.5	nd	nd	nd	nd	nd	nd	CML, AML, and histiocytic Ly	nd
Ryczek/2013	1	CR (Poland)	nd	nd	nd	nd	nd	nd	nd	nd	CML	nd

Schnack/1954	1	CR (Austria)	53 F	8	D	nd	nd	nd	nd	nd	MM	nd

Senel/2006	1	CR (Turkey)	65 F	0	D	Scl70	L, K	No	nd	Weakness, sweating, and weight loss	CML	nd

Shvidel/2002	1	CS (Israel)	71 F	nd	nd	nd	nd	nd	nd	nd	T large granular lymphocytic leukemia	nd

Siau/2011	5	CS (68 UK pts)	nd	0 (case of PC)	L	nd	nd	nd	nd	nd	MM (2), diffuse large B-NHL, thyroid NHL, solitary IgM PC	nd

Sidi/1990	2	CS (Israel)	47 M, 77 M	20; 11	L	nd	CREST	No	nd	Generalized lymphoadenopathy	B-CLL	Alive up to 2 years; death for bronchopneumonia and paralytic ileus

Sugai/1987	1	CR (Japan)	67 F	11	D	ANA	E, L	Yes	nd	Parotid swelling and generalized lymphadenopathy	IIIb-staged NHL	Death after 3 COPP cycles for complicating interstitial pneumonitis

Suzuki/1994	1	CR (Japan)	68 M	3	D	ANA	nd	No	nd	Gait disturbance, anemia, and hemorrhagic stroke	Brain diffuse large B-NHL	Death for pneumonitis during BACOPP chemotherapy

Szekanecz/2008	3	CS (218 Hungarian pts)	53; 67; 69 F	2; 1.9; 0.7	D	Scl70;	L-H-E; none; L-H-K-E	nd	nd	nd	(2) b-CLL; (1) chronic small lymphocytic B NHL	Surviving > 5 years

Talbott/1979	2	CS (USA)	64 M; 73 M	<1; 10	L; D	nd	None; L-H	Probable	Pt number 1 coal miner	Backache; generalized weakness	MM	Rapid deterioration and death

Vettori/2010	1	CR (Italy)	45 F		Sine sclerod.					Progressive weight loss	Gastric B-cell Ly	

Watanabe/1994	1	CR (Japan)	44 F	nd	nd	nd	nd	nd	nd	Leukocytosis, thrombocytosis	CML	CML remission and SSc improvement with therapy

William/2011	1	CR (USA)	61 M	30	L	ACA	E, L	No	nd	Thrombocytopenia, cervical adenopathy	Small lymphocytic B-NHL	Remission with FCR

Wooten/1998	1	CR (USA)	nd	3	L		CREST	nd	Porphyria c.t.	nd	CML	nd

Yamamoto/2005	1	CR (Japan)	72 M	5	D	Scl70	L	nd	nd	Multiple lymphoadenopathy	Angioimmunobl. T cell Ly with EBV-assoc. B cell lymphoprol. dis.	Died 6 months after CHOP therapy because of sepsis, initially improved

*Present study*	2	CR (Italy)	37 M; 72 F	2; 28	D; L	SSA/SSB ACA	E, L; CREST + L	Yes; no	nd; previous breast cancer	Weakness, sweating, and weight loss; asymptomatic	Diffuse large B-NHL; marginal B-NHL	Died few months after during R-CHOP therapy; lung lobe resection and remission

Total	130 pts											

The table included all the case reports and the cohort studies that reported cases of haematological malignancies in the course of SSc [4–67]. Pts = patients; type of study: CR = case report; CS = case series/cohort studies; RS = registry studies; skin subset: D = diffuse, L = limited; serology: ACA = anticentromere, Scl70 = anti-topoisomerase I, ANA = specific antinuclear autoantibodies; organ involvements: K = kidney, L = lung, H = heart, E = esophagus; MGUS = monoclonal gammopathy of undetermined significance; CREST = former acronym for limited SSc form including calcinosis, Raynaud phenomenon, esophageal dysmotility, sclerodactyly, and telangiectasia; CLL = chronic lymphocytic leukemia; CML = chronic myelogenous leukemia; AML = acute myelogenous leukemia; MM = multiple myeloma; PC = plasmacytoma; NHL = non-Hodgkin lymphoma; CMML = chronic myelomonocytic leukemia; Ly = lymphoma; HL = Hodgkin lymphoma; MTX = methotrexate; (R-)CHOP = chemotherapic regimen for NHL; FCR = chemotherapic regimen with fludarabile, cyclophosphamide, and rituximab; BMT = bone marrow transplantation; (BA)COPP = bleomycin, adriamycin, cyclophosphamide, vincristine, procarbazine, and prednisone; MOPP = mustine, vincristine, procarbazine, and prednisone; HU = hydroxyurea; VAD = vincristine, doxorubicin, and dexamethasone.
